# Is Spirometry a Sufficient Test for Assessing Respiratory Function after Lung Resection?

**DOI:** 10.3390/curroncol31070295

**Published:** 2024-07-11

**Authors:** Damian Wnuk, Tomasz Marjański, Bartłomiej Tomasik, Joanna Żuralska-Wnuk, Witold Rzyman

**Affiliations:** 1 Division of Physical Therapy, Faculty of Health Sciences with the Institute of Maritime and Tropical Medicine, Medical University of Gdańsk, 80-210 Gdańsk, Poland; joanna.zuralska-wnuk@gumed.edu.pl; 2 Department of Thoracic Surgery, Faculty of Medicine, Medical University of Gdańsk, 80-210 Gdańsk, Poland; tomasz.marjanski@gumed.edu.pl (T.M.); witold.rzyman@gumed.edu.pl (W.R.); 3 Department of Oncology and Radiotherapy, Faculty of Medicine, Medical University of Gdańsk, 80-210 Gdańsk, Poland; bartlomiej.tomasik@gumed.edu.pl

**Keywords:** lung cancer, rehabilitative and perioperative care, respiratory function

## Abstract

Background: The prediction of postoperative functional status in non-small cell lung cancer patients based on preoperative assessment of physical and respiratory capacity is inadequate based on recent RCTs. Material and methods: Prospectively collected spirometry data and the six-minute walk test results of 57 patients treated with lobectomy for non-small cell lung cancer were analyzed. The tests were performed before surgery, and 30 and 90 days after lobectomy. All patients underwent a respiratory functional and physical capacity assessment. Results: All 57 patients underwent lobectomy. Before surgery, mean FEV1 was 2.4 ± 0.7 L, corresponding to %FEV1 of 88.3 ± 17.3%. The mean absolute and expected 6MWT distance was 548 ± 74.6 m and 108.9 ± 14.5%, respectively. At the first postoperative evaluation 30 days after surgery, FEV1 and %FEV1 decreased significantly by an average of 0.5 ± 0.3 L and 15.1 ± 10.7%, while 6MWT and expected 6MWT decreased minimally by an average of 1.0 m and 0.8%, respectively. Three months after lobectomy, FEV1 and %FEV1, compared with the initial assessment, decreased by an average of 0.3 ± 0.3 l and 7.8 ± 10.0%, while 6MWT and its expected score increased to 564.6 ± 84.6 m and 112.8 ± 15.8%, respectively. Conclusions: After lobectomy, FEV1 decreased slightly and less than expected, while 6MWT increased proportionally compared to the preoperative evaluation.

## 1. Introduction

Lung resection is the gold standard procedure for patients with stage I–IIIA non-small cell lung cancer (NSCLC). Respiratory function and physical capacity assessments are essential parts of the pre-treatment workup, allowing prediction of postoperative functional status. Spirometry is a widely accepted method of respiratory function testing, with a special emphasis on forced expiratory volume (FEV1) value. Lung resection reduces respiratory surface area and can lead to a decrease in spirometric parameters—up to 25% of preoperative—especially when lobectomy, the standard treatment for lung cancer patients, is performed [1]. Surgical treatment indirectly leads to a decline in physical activity due to decreased respiratory capacity. Physical activity is one of the components of satisfaction assessment and is an integral part of patients’ quality of life assessment [2,3,4]. The most objective method of physical capacity assessment is the Cardiopulmonary Exercise Test (CPET). In addition, the Stair Climbing Test (SCT), Shuttle Walking Test (SWT), and Six Minute Walking Test (6MWT) are widely used as low-tech methods [5,6,7].

Two randomized controlled trials (RCTs) evaluating long-term outcomes of surgical treatment were published in 2022 and 2023. These studies evaluated whether sublobar resection for stage IA NSCLC was non-inferior to lobectomy in terms of long-term survival [8,9]. In addition to primary endpoints, both studies assessed the loss of respiratory function measured using spirometry, with a focus on FEV1 values in both arms of the study. Noteworthy, lower-than-expected loss of respiratory function after lobectomy was reported in both RCTs. It is therefore crucial to answer whether spirometry is a reliable test for predicting respiratory function after lung resection. In this prospective study, we compare spirometry with 6MWT results performed in patients before and after lobectomy. We also report the relationship between dynamically changing spirometric values and patients’ physical fitness as measured by the distance covered in the 6MWT in the early postoperative period.

## 2. Materials and Methods

**Figure 1:**
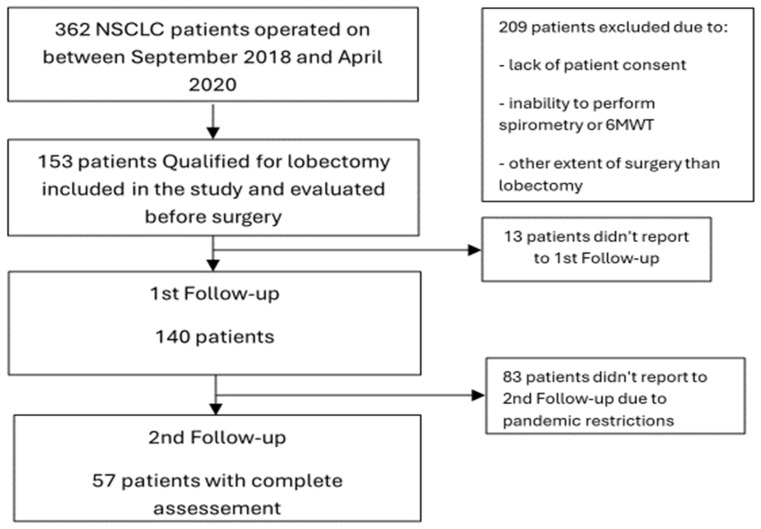
Flowchart showing patient enrollment.

A retrospective analysis of prospectively collected data in a group of patients treated for lung cancer between September 2018 and April 2020 in a tertiary cancer center was conducted. A total of 153 consecutive patients who underwent lobectomy and agreed to participate in the study were included, and 57 patients were finally enrolled (Figure 1).

Such a significant reduction in the study group was associated with restrictions imposed due to the COVID-19 pandemic at the time our study was conducted. Preoperative qualification for surgery was done according to the current treatment indications in early NSCLC and included radiological examinations, respiratory functional assessment, cardiological evaluation, and physical capacity assessment of each patient. The preoperative characteristics of the study group are shown in Table 1.

In all patients, spirometry and 6MWT were performed on the same day and at the same intervals; one day before, then 30 and 90 days after surgery. The 6MWT test was performed under the same conditions, in a 33 m corridor by two trained physiotherapists and with an on-call physician available. Spirometry was performed before the exercise test by qualified staff using a CareFusion_JAEGER spirometer (Model: MasterScreen Pneumo/Rhino/APS PRO), CareFusion Germany 234 GmbH, Hoechberg, Germany. Both postoperative evaluations of the studied parameters were combined with routine postoperative control visits in the outpatient department. The inability to perform spirometry and/or the 6MWT was an exclusion criterion. Based on “ERS/ESTS clinical guidelines on fitness for radical therapy in lung cancer patients” we calculated postoperative FEV1, applying the recommended calculations: predicted postoperative -FEV_1_ = preoperative FEV_1_ × (1 − a/b), where a is the number of unobstructed segments to be resected and b is the total number of unobstructed segments [5]. We performed lobectomy in 57 patients, in whom 216 of 1083 segments were resected. The formula used predicted an average postoperative FEV1 loss of 19.9%. After surgery, all patients received standard medical, nursing, psychological, and physiotherapeutic support. From the first postoperative day, physiotherapeutic management was tailored to the individual patient’s needs and abilities. The physiotherapeutic management scheme is shown in Table 2.

Statistical analysis: Continuous variables are presented as means along with standard deviations, and nominal variables are presented as counts along with percentages. Qualitative variables were compared using the chi-squared test. The normal distribution of continuous variables was verified using the W Shapiro–Wilk test. Continuous variables were analyzed using the *t*-test (paired or unpaired) or Mann–Whitney U test, depending on the distribution of the data. Correlations were assessed using Spearman’s rank correlation. Differences between multiple measurements were tested using ANOVA with repeated measures. All statistical analyses were performed using Statistica version 13.3 (TIBCO, Palo Alto, CA, USA). Values of *p* < 0.05 were considered statistically significant.

Sample size calculation: the primary aim of this study was to compare preoperative and postoperative respiratory functions using spirometry and the 6MWT in patients undergoing lobectomy for NSCLC. The effect size was derived from our previous studies—we assumed a moderate effect size for spirometric changes (mean difference of 0.2 L and standard deviation of differences of 0.5 L). Based on paired *t*-tests and assuming α = 0.05 and β = 0.2, we calculated that at least 52 patients are needed to perform this study. This number was increased by 10% to account for possible dropouts, resulting in the final number of 57 patients.

## 3. Results

**Figure 2:**
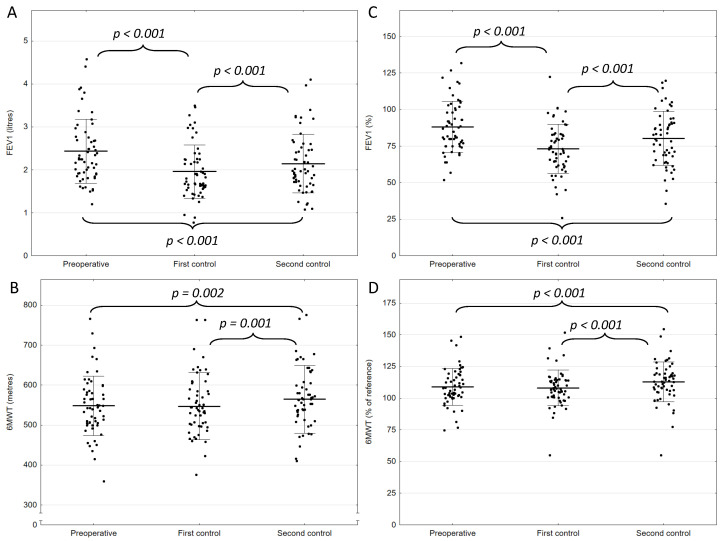
Results of pre-and postoperative tests of lung function and physical capacity. The whisker plots show the results of (**A**) FEV1 in liters, (**B**) 6MWT in meters, (**C**) FEV1 in %, and (**D**) 6MWT as % of reference at three different time points (preoperative, during the first control three weeks after the surgery, and during the second control three months after the surgery). Individual measurements are presented as dots, the mean value is presented as a horizontal line, and whiskers represent the standard deviations.

**Figure 3:**
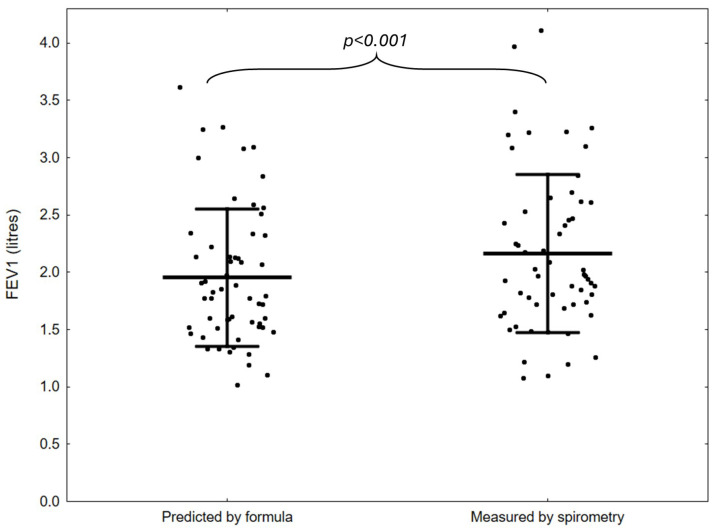
FEV1 values predicted by the formula and measured by spirometry, three months after surgery, compared using paired *t*-tests. Individual measurements are presented as dots, the mean value is presented as a horizontal line, and whiskers represent the standard deviations.

All patients underwent lobectomy. The majority of patients (n = 39, 68%) underwent video-assisted thoracic surgery (VATS) and 18 patients (32%) underwent thoracotomy (Table 1). There were no deaths within 90 days post-operation. In the second stage of the assessment, three weeks after surgery, there was a significant reduction in the values of both FEV1 and FEV1%: FEV1 by 0.5 ± 0.3 L and FEV1% by 15.1 ± 10.7% (*p* < 0.001 for both comparisons). Three months after the lung resection, the assessed spirometric parameters improved: FEV1 dropped by a mean of 0.3 ± 0.3 L compared to the preoperative assessment, and FEV1% decreased by an average of 7.8 ± 10.0%. Differences between these two time points were significant (*p* < 0.001) for both FEV1 and FEV1% (Figure 2A,B). We did not observe significant differences in preoperative FEV1 values between patients who underwent VATS and thoracotomy (2.5 ± 0.8 vs. 2.3 ± 0.8 L, respectively; *p* = 0.529). Repeated measures ANOVA did not show differences between the groups in the other time points (*p* = 0.654). FEV1 loss, expressed in liters compared to preoperative values, was 17.9 ± 13.9% of volume three weeks after surgery and 10.9 ± 12.2% after 90 days. The actual mean values measured by spirometry were significantly higher than the values predicted by the formula, i.e., 2.16 ± 0.69 L vs. 1.95 ± 0.60 L (*p* < 0.001), respectively (Figure 3).

The mean 6MWT distance at the first postoperative examination was not significantly different from the preoperative examination in terms of both absolute values (548.0 ± 74.6 m vs. 547.0 ± 83.5 m; *p* = 0.623) and expected values (108.9 ± 14.5% vs. 108.1 ± 14.0%; *p* = 0.471) calculated using the Enright and Sherill equation [10]. At the time of the third 6MWD test, the average distance the patient walked was 564.6 ± 84.6 m, representing an average of 112.8 ± 15.8% of the expected value. The 6MWT score showed a significant increase over time, with measurements taken after 3 months demonstrating a notable rise compared to the baseline and the values recorded 3 weeks into the study (Figure 2C,D). We did not observe significant differences in preoperative 6MWD values between patients who underwent VATS and thoracotomy (556.5 ± 73.7 vs. 527.8 ± 75.9 m, respectively, *p* = 0.181). Repeated measures ANOVA did not show differences between the groups in the other time points (*p* = 0.918).

FEV1 (in liters) and 6MWD (in meters) served as metrics to evaluate the effects of treatment, revealing a correlation between their absolute values at each measurement point (Table 3). The calculated expected values of both parameters, expressed as a percentage, only correlated in the postoperative phase, with no such correlation observed preoperatively.

## 4. Discussion

The long-term outcomes of overall survival and recurrence-free survival of patients with NSCLC treated with lobectomy and sublobar resection are similar [8,9]. Permanent loss of lung function was demonstrated by spirometry in both approaches, as well as in two recently published RCTs. Interestingly, loss after lobectomy appeared to be much lower than expected based on the mathematical calculation [8,9]. We hypothesized that the ERS/ESTS guidelines suggesting the use of a given predicted postoperative FEV1 calculation are not applicable in the real world, as the JCOG and CALBG studies also suggest by overestimating lung function loss [8,9], and that the 6MWT provides additional value in predicting the patient’s postoperative functional status. In our study, we confirmed these findings. FEV1 after lobectomy is much better than predicted by the mathematical calculation used to predict postoperative respiratory capacity [5]. This study also shows that the 6MWD score after lobectomy is better than before surgery, which indirectly supports the thesis that the difference in respiratory capacity after lobectomy as well as segmentectomy, i.e., limited resection, is non-significant.

The reduction in spirometric values and a difference between lobectomy and segmentectomy in the early postoperative period was demonstrated by Xu et al. [11]. Similarly, Varela et al. showed a loss of FEV1 in the immediate period after lobectomy. In addition, they found that the FEV1 loss was significantly greater than that estimated based on preoperative parameters [12]. These results contradict ours and the cited results of the two RCTs. However, in order to determine the permanent loss of spirometric parameters, we should avoid evaluation immediately after surgery, when the greatest limitation for patients is postoperative pain. Better results during follow-up after 90 days compared to follow-up after 30 days demonstrate the decreasing influence of surgery-related factors.

Efficient respiratory function is crucial for physical performance, with a patient’s capacity for physical exertion serving as a key indicator of fitness. The 6MWT is a valuable adjunct to preoperative assessment and we used it in previous studies as an independent test to stratify the level of risk of postoperative complications [13,14]. In the present study, we applied the 6MWT as a validation test for FEV1 values in the postoperative period and found even lesser loss than the assessed using spirometry. The significant correlation between spirometric parameters and 6MWD in the postoperative period confirms the observations of Grenger et al., who found a similar level of correlation between these parameters [6]. Despite the fact that the 6MWT is a submaximal effort-based test that is dependent on patient involvement, it should be considered as an additional element in the functional assessment of treatment effects. Thus, it serves as a simple indirect test for the assessment of the cardiorespiratory capacity of patients with respiratory failure [15,16]. The potential weakness of this study is the fact that based on preoperative functional assessment, spirometry results (%FEV1 > 88%), and an average walk test distance of over 500m, our cohort may be classified as a low-risk group [5,6,13,14,17]. Evaluation of high-risk candidates for surgical treatment, who constitute a minority of our study group, remains difficult and requires a multidisciplinary approach at each stage of treatment. Decreased lung function is one of the risk factors for the development of postoperative complications, thus precise assessment of respiratory parameters is crucial. Exercise testing, including 6MWT, has significant additive value in predicting the risk of complications [17,18]. The occurrence of respiratory complications is associated with poorer long-term outcomes [19]. Surgical techniques can also affect postoperative lung function outcomes. Many reports have emphasized the superiority of VATS over open thoracotomy in many different aspects, including long-term outcomes, i.e., overall survival [20,21,22,23,24,25,26]. We conducted a similar comparison in our study and did not observe significant differences; however, this may be due to the small sample size and the limited population of patients undergoing thoracotomy. Another element that can affect postoperative spirometry results is the presence of emphysema in the removed lung parenchyma. In such a situation, the loss of lung function may be relatively less expressed and sometimes even improved.

Despite the fact that there is a permanent decrease in spirometric parameters after lung resection, after the operation it is possible to return or even improve the level of physical performance presented before surgery. This is indicated by the results of the 6MWT. This observation corroborates an earlier study by Brocki et al. who demonstrated a permanent reduction in FVC and FEV1 values in a study group of patients but found a return to preoperative performance levels as measured by the 6MWD six months after pulmonary resection [27]. In addition, they indicated a sustained reduction in SpO2 levels during the walk test compared to preoperative results. Desaturation during exercise is another indicator of cardiopulmonary fitness that has also been reported [28,29]. Another pulmonary function test performed in preoperative evaluation is DLCO (diffusion lung capacity for carbon monoxide) [5,6], which can be regarded as a potential limitation of this study. The time required to return to baseline performance levels described in various studies is 3–6 months [7,27,30]. In the case of our study, patients improved their 6MWD scores over preoperative values in the long term after surgery. This may have been associated with changes in habits and lifestyle after treatment, i.e., increased physical activity levels as a result of intensive postoperative rehabilitation, changes in dietary habits, or smoking cessation in smokers. We found no significant differences in all analyzed parameters between men and women.

## 5. Conclusions

In lung cancer patients who were operated on, the FEV1 measurements 30 and 90 days post-lobectomy were higher than the pre-surgery-predicted postoperative FEV1 (ppoFEV1) values, indicating a lower-than-expected loss. The 6MWT is a valuable adjunct to spirometry testing in predicting a patient’s postoperative performance.

## Figures and Tables

**Table 1:** Patients’ characteristics.

	Preoperative Assessment	Assessment at 1st Follow-Up	Assessment at 2nd Follow-Up
Age (years)	65.2 ± 9.0		
Postoperative stay (days)	6.3 ± 3.3		
BMI	27.1 ± 4.7		
Male/Female	n = 33/24		
VATS	n = 39 (68%)		
Preoperative FEV1/FVC	0.71 ± 0.09		
6MWD (m)	548.0 ± 74.6	547.0 ± 83.5	564.6 ± 84.6
Percentage of due average 6MWT (%)	108.9 ± 14.5	108.1 ± 14.0	112.8 ± 15.8
FEV1 (L)	2.4 ± 0.7	2.0 ± 0.6	2.1 ± 0.7
%FEV1 (%)	88.3 ± 17.3	73.2 ± 16.9	80.4 ± 18.5
FVC (L)	3.5 ± 1.0	3.0 ± 0.9	3.3 ± 0.9
%FVC (%)	100.7 ± 16.7	86.4 ± 20.5	97.7 ± 19.3
Smoking status:			
- ever smoked		n = 52 (91%)	
- never smoked		n = 5 (9%)	
Extent of surgery (number of lung segments removed)			
Lower right lobectomy (5/19)		n = 9 (16%)	
Upper right lobectomy (3/19)		n = 23 (40%	
Lower left lobectomy (4/19)		n = 17 (30%)	
Upper left lobectomy (5/19)		n = 6 (10.5%)	
Middle lobectomy (2/19)		n = 2 (3.5%)	

Data presented as N (%) or mean with standard deviation (SD). BMI = body mass index, VATS = video-assisted thoracic surgery, 6MWT/6MWD = six-minute walking test/distance, FEV1 = forced expiratory volume in 1 s, FVC = forced volume capacity.

**Table 2:** Scheme of postoperative physiotherapeutic management.

DAY I	breathing exercises performed in a normal breathing pattern support for effective expectoration active exercises of the lower and upper extremities within an accessible, pain-free range of mobility verticalization moving within the patient room and hospital corridor with the assistance of a physiotherapist	Repeated, independent repetition of learned exercises and therapeutic techniques
CONSECUTIVE DAYS AFTER SURGERY	continuation of improvement from the first day the level of difficulty of the exercises and the ranges of mobility of the involved joints were increased increased intensity of walking (pace, frequency, self-repetition) walking on stairs with the physiotherapist’s assistance	
Education on the need for continued improvement and maintenance of fitness in the post-hospital period		

**Table 3:** Correlation matrix of 6MWT and FEV1.

	FEV 1—Preoperative	FEV 1—First Control	FEV 1—Second Control
6 MWT—preoperative	r = 0.39	r = 0.34	r = 0.42
	*p* < 0.001	*p* < 0.001	*p* = 0.001
6MWT—first control	r = 0.42	r = 0.42	r = 0.51
	*p* < 0.001	*p* < 0.001	*p* < 0.001
6 MWT—second control	r = 0.43	r = 0.47	r = 0.51
	*p* = 0.001	*p* < 0.001	*p* < 0.001

## Data Availability

The raw data supporting the conclusions of this article will be made available by the authors upon request.
